# TNF-α promotes cerebral pericyte remodeling *in vitro*, via a switch from α1 to α2 integrins

**DOI:** 10.1186/1742-2094-10-33

**Published:** 2013-03-01

**Authors:** Ulrich Tigges, Amin Boroujerdi, Jennifer V Welser-Alves, Richard Milner

**Affiliations:** 1 Department of Molecular and Experimental Medicine, The Scripps Research Institute, 10550 North Torrey Pines Road, La Jolla, CA, 92037, USA

**Keywords:** Pericyte, Adhesion, Migration, Extracellular matrix, Integrin, Vascular remodeling

## Abstract

**Background:**

There is increasing evidence to suggest that pericytes play a crucial role in regulating the remodeling state of blood vessels. As cerebral pericytes are embedded within the extracellular matrix (ECM) of the vascular basal lamina, it is important to understand how individual ECM components influence pericyte remodeling behavior, and how cytokines regulate these events.

**Methods:**

The influence of different vascular ECM substrates on cerebral pericyte behavior was examined in assays of cell adhesion, migration, and proliferation. Pericyte expression of integrin receptors was examined by flow cytometry. The influence of cytokines on pericyte functions and integrin expression was also examined, and the role of specific integrins in mediating these effects was defined by function-blocking antibodies. Expression of pericyte integrins within remodeling cerebral blood vessels was analyzed using dual immunofluorescence (IF) of brain sections derived from the animal model of multiple sclerosis, experimental autoimmune encephalomyelitis (EAE).

**Results:**

Fibronectin and collagen I promoted pericyte proliferation and migration, but heparan sulfate proteoglycan (HSPG) had an inhibitory influence on pericyte behavior. Flow cytometry showed that cerebral pericytes express high levels of α5 integrin, and lower levels of α1, α2, and α6 integrins. The pro-inflammatory cytokine tumor necrosis factor (TNF)-α strongly promoted pericyte proliferation and migration, and concomitantly induced a switch in pericyte integrins, from α1 to α2 integrin, the opposite to the switch seen when pericytes differentiated. Inhibition studies showed that α2 integrin mediates pericyte adhesion to collagens, and significantly, function blockade of α2 integrin abrogated the pro-modeling influence of TNF-α. Dual-IF on brain tissue with the pericyte marker NG2 showed that while α1 integrin was expressed by pericytes in both stable and remodeling vessels, pericyte expression of α2 integrin was strongly induced in remodeling vessels in EAE brain.

**Conclusions:**

Our results suggest a model in which ECM constituents exert an important influence on pericyte remodeling status. In this model, HSPG restricts pericyte remodeling in stable vessels, but during inflammation, TNF-α triggers a switch in pericyte integrins from α1 to α2, thereby stimulating pericyte proliferation and migration on collagen. These results thus define a fundamental molecular mechanism in which TNF-α stimulates pericyte remodeling in an α2 integrin-dependent manner.

## Introduction

Pericytes are vascular mural cells that lie in close proximity to endothelial cells of capillaries, arterioles, and venules [1,2]. Pericytes are regularly positioned along cerebral microvessels, and ultrastructural studies have shown that they are located within the abluminal vascular basal lamina that surrounds vessels [3]. Pericytes are crucial regulators of vascular development, stability, and remodeling [4], and increasing evidence suggests that they also regulate capillary blood flow [5,6]. One area currently attracting great interest is the role of pericytes in vascular remodeling. The current view is that pericytes act as central regulators of angiogenesis, through their ability to stabilize or destabilize microvessels [7,8]. According to this view, pericytes promote vessel stability by maintaining close adhesive contacts with both endothelial cells and the underlying ECM, thus locking the vascular components into place. At an early stage of the angiogenic program, pericytes undock from endothelial cells, migrating within the ECM-rich basal lamina [9]. This leads to endothelial cells breaking connections, both with each other and with the underlying basal lamina, in order to migrate and proliferate, and to sprout new blood vessels. Upon completion of endothelial remodeling, pericytes migrate back to regain contact with endothelial cells, thereby stabilizing newly formed vessels. This important role for pericytes in vascular remodeling is best illustrated by the finding that mutant mice lacking platelet-derived growth factor beta (PDGF-B) or the PDGF-β receptor fail to show efficient pericyte coverage of blood vessels, resulting in perinatal lethality due to leaky dysfunctional blood vessels [10,11].

The basal lamina of cerebral blood vessels comprises a number of different ECM proteins and proteoglycans, the precise make-up of which varies with the vessel-maturation state. The basal lamina of mature vessels is comprised of three major constituents: collagen IV, laminins, and heparan sulfate proteoglycan (HSPG) [12-14]. In addition, immature vessels of the developing CNS and those undergoing remodeling in the adult CNS also contain increased levels of fibronectin and vitronectin, which are downregulated upon vessel maturation [15,16]. Broadly speaking, the ECM influences many aspects of cell behavior, including cell proliferation, migration, differentiation, and stabilization [17,18]. These effects are mediated by the ECM receptors, integrins, which are expressed at the cell surface as αβ heterodimers, of which the β1 class is the major type [19,20]. In a number of studies, we have highlighted a role for the remodeling protein fibronectin in driving cerebral angiogenesis after cerebral hypoxia or ischemia. In these models, angiogenic cerebral vessels show strong upregulation of fibronectin and the fibronectin receptor, α5β1 integrin [21,22]. Furthermore, using endothelial-specific deletion of the α5 integrin, we showed previously that α5β1 integrin plays an important role in promoting endothelial cell proliferation at an early stage of the angiogenic response [23].

As pericytes lie within the basal lamina ECM of cerebral microvessels [3], it seems likely that pericytes also respond to environmental cues provided by the ECM. Because little is currently known about the influence of the ECM on pericyte behavior, the aim of this study was to address the following questions: 1) how is cerebral pericyte adhesion, proliferation, and migration influenced by the different ECM constituents present in the vascular basal lamina; 2) which integrins do pericytes express; 3) how do cytokines regulate pericyte remodeling state and expression of integrins; and 4) are any of the identified integrins required for pericyte remodeling?

## Materials and methods

### Animals

The studies described were reviewed and approved by the Scripps Research Institute (TSRI) Institutional Animal Care and Use Committee. All cell cultures were obtained from C57Bl/6 mice, which were maintained under pathogen-free conditions in the closed breeding colony of TSRI.

### Experimental autoimmune encephalomyelitis

Experimental autoimmune encephalomyelitis (EAE) was induced using a commercial protocol and materials (Hooke Laboratories, Lawrence, MA, USA). Briefly, C57Bl/6 female mice, 8 to 10 weeks old, were immunized with 100 μl of 1 mg/ml MOG_33-35_ peptide emulsified in complete Freund’s adjuvant (CFA) containing 2 mg/ml *Mycobacterium tuberculosis* by subcutaneous injection in both the base of the tail and upper back. In addition, on days 0 and 1, mice also received an intraperitoneal injection of 200 ng pertussis toxin. Control mice received CFA not containing the MOG peptide. This protocol leads to robust induction of clinical EAE on days 12 to 14 after immunization. Animals were monitored daily for clinical signs and scored as follows: 0, no symptoms; 1, flaccid tail; 2, paresis of hind limb; 3, paralysis of hind limbs; 4, quadriplegia; 5, death. At 21 days post-immunization, corresponding to the acute symptomatic stage of disease, mice were euthanized by intraperitoneal injection of sodium pentothal.

### Cell culture

Pure cultures of mouse brain endothelial cells (BECs) or pericytes were prepared as previously described [24,25]. Briefly, brains were removed from 8 week-old C57Bl/6 mice, minced, dissociated for 1 hour in papain and DNase I, centrifuged through 22% BSA to remove myelin, and endothelial cells cultured in endothelial cell growth media (ECGM), consisting of Hams F12 supplemented with 10% FBS, heparin, ascorbic acid, L-glutamine, penicillin/streptomycin (all from Sigma Chemical Co., St. Louis, MO, USA) and endothelial cell growth supplement (ECGS) (Upstate Cell Signaling Solutions, Lake Placid, NY, USA), on six-well plates coated with type I collagen (Sigma Chemical Co.). To obtain BECs, puromycin (4 μg/ml; Alexis GmbH, Grunberg, Germany) was included in the culture media on days 1 to 3 to remove contaminating cell types. Endothelial cell purity was >99% as determined by flow cytometry with CD31. For all experiments, BECs were used only for the first passage.

Pericytes were obtained using the same approach, except that the puromycin step was omitted. The pericyte cultures were grown in ECGM, with the medium changed every 3 days. On reaching confluency, cultures were harvested with trypsin and passaged. During the first two passages, pericyte cultures were grown in ECGM, but on the third passage, they were switched to pericyte medium (PCM; ScienCell Research Laboratories, Carlsbad, CA, USA) containing 2% FBS. In previous studies we found that, using this approach, cultures of pericytes become highly purified after the third passage, at which point these cultures are more than 99% pericytes as determined by expression of the pericyte marker NG2 and the PDGF-β receptor, and contain less than 1% of contaminating endothelial cells (CD31), astrocytes (glial fibrillary acidic protein; GFAP), or microglia (Mac-1), as determined by fluorescent immunocytochemistry [25]. All studies were performed on pericytes at passages 4 to 8. Pericytes were expanded in PCM containing 2% FBS, but all functional assays were performed in serum-free DMEM containing N1 supplement, L-glutamine, and penicillin/streptomycin (all from Sigma Chemical Co.).

### Cytokine treatment and antibodies

To investigate the influence of cytokines on pericyte behavior and expression of integrin subunits, pericytes were cultured on collagen I in the presence of 20 ng/ml basic fibroblast growth factor (bFGF; Invitrogen Corp., Carlsbad, CA, USA), 20 ng/ml platelet derived growth factor (PDGF-B) 2 ng/ml transforming growth factor (TGF)-β1 10 ng/ml tumor necrosis factor (TNF)-α, or 10 ng/ml vascular endothelial growth factor (VEGF) (all R&D Systems, Minneapolis, MN, USA). These concentrations were selected based on the findings of previous studies [26,27]. The following monoclonal antibodies (BD Pharmingen, La Jolla, CA, USA) were used: monoclonal antibodies reactive for the integrin subunits α1 (clone Ha31/8), α2 (clone Ha1/29), α4 (clone MFR4.B), α5 (clone 5H10-27 (MFR5), α6 (clone GoH3), β1 (clone Ha2/5), and Mac-1 (clone M1/170); CD31 (clone MEC13.3); and isotype control antibodies: rat anti-KLH (A110-2) and hamster anti-TNP-KLH (G235-1). Other antibodies used in this study included Cy3-conjugated anti-GFAP (Sigma Chemical Co.) and rabbit anti-NG2 and anti-PDGF-β receptor antibodies (both kindly provided by Dr William Stallcup, Sanford-Burnham Medical Research Institute, La Jolla, CA, USA).

### Flow cytometry

Integrin expression by BECs and pericytes (treated with different cytokines for 2 days) was examined as described previously [26]. Briefly, cells were removed from the six-well culture plates, and cell-surface expression of the integrin subunits α1, α2, α4, α5, α6, or β1 was analyzed by flow cytometry using phycoerythrin (PE)-conjugated monoclonal antibodies (all BD Pharmingen). The fluorescent intensity of the labeled cells was analyzed with a flow cytometer (FACScan; Becton Dickinson, San Diego, CA, USA), with 10,000 events captured for each condition. In each experimental condition, the mean fluorescent intensity was compared with the control (no factor) condition, and expressed as the percentage change relative to control. Each experiment was repeated a minimum of four times.

### Cell-adhesion assays

Adhesion assays were performed as described previously [28]. Briefly, substrates were prepared by coating the central area of glass coverslips in 24-well plates (Nunc; BD Biosciences, San Jose, CA, USA) with 25 μl of ECM solution (10 μg/ml of collagen I, collagen IV, fibronectin, HSPG, or laminin-1; all from Sigma Chemical Co.) for 2 hours at 37°C. Substrates were washed twice before addition of cells. Pericytes were prepared as described above, centrifuged, and re-suspended in N1 serum-free media, then 2,000 cells were applied to the substrates in a 25 μl drop and incubated at 37°C for 1, 4, or 8 hours. In function-blocking experiments, antibodies were included at 5 μg/ml. The assay was stopped by adding 1 ml of DMEM and washing off any loosely attached cells. Attached cells were fixed in 4% paraformaldehyde in PBS for 20 minutes, and stored in PBS. Adhesion was quantified under phase microscopy by counting all attached cells within five fields of view per condition. In each experiment, each condition/time-point was performed in duplicate.

### Proliferation assays

Glass coverslips were coated with the ECM substrates as described above, and pericytes plated out in serum-free N1 medium. Cytokines and/or integrin-blocking antibodies were included at the time of plating. The following morning, pericytes were incubated for 3 hours with bromodeoxyuridine (BrdU; Invitrogen Corp.), fixed in acid/alcohol, and analyzed by immunofluorescence for BrdU incorporation, in accordance with the manufacturer’s instructions. BrdU-positive cells were expressed as the percentage of total cells (Hoechst staining).

### Migration assays

Pericyte migration was quantified using the scratch assay. Pericytes were plated into ECM-coated wells of a 24-well plate, and cultured in PCM. Upon reaching confluence, vertical and horizontal scratches were made in the monolayer using a 1 ml pipette tip. The PCM and cell debris were removed, and replaced with serum-free N1 medium (cytokines and/or integrin-blocking antibodies were included at this time), and the scratch width was recorded for all samples. The new width of the scratch was recorded 16 hours later, and the distance of cell migration calculated. In each experiment, each condition was performed in duplicate.

### Immunohistochemistry and analysis

Immunohistochemistry was performed as described previously [28], on 10 μm frozen sections of cold PBS-perfused brain, using monoclonal antibodies specific for the integrin subunits α1 (clone Ha31/8) and α2 (clone Ha1/29), and the pericyte marker NG2. Secondary antibodies used included anti-hamster Alexa Fluor 488 (Invitrogen Corp.) and anti-rabbit Cy3 (Jackson Immunoresearch, Baltimore, PA, USA). Images of brain sections were taken using a ×20 objective on a microscope (Imager M1.m; Zeiss, Thornwood, NY, USA). Three images were taken, and the number of integrin-positive vessels per field of view recorded for each section per subject.

### Statistical analysis

All results represent the mean ± SEM of four experiments, except for immunohistochemistry, which was performed with three different animals per condition. The Student’s *t*-test was used to analyze the results of the proliferation assays and the immunohistochemistry, while the paired Student’s *t*-test was used to analyze the results for flow cytometry, and the cell-adhesion and migration assays. For all tests, *P*<0.05 was defined.

## Results

### Extracellular-matrix constituents differentially regulate cerebral pericyte behavior

To investigate how pericyte behavior is influenced by the different ECM molecules present in the vascular basal lamina, pure populations of cerebral pericytes were cultured in serum-free N1 medium on glass coverslips coated with collagen I, collagen IV, fibronectin, HSPG, or laminin-1.

**Figure 1:**
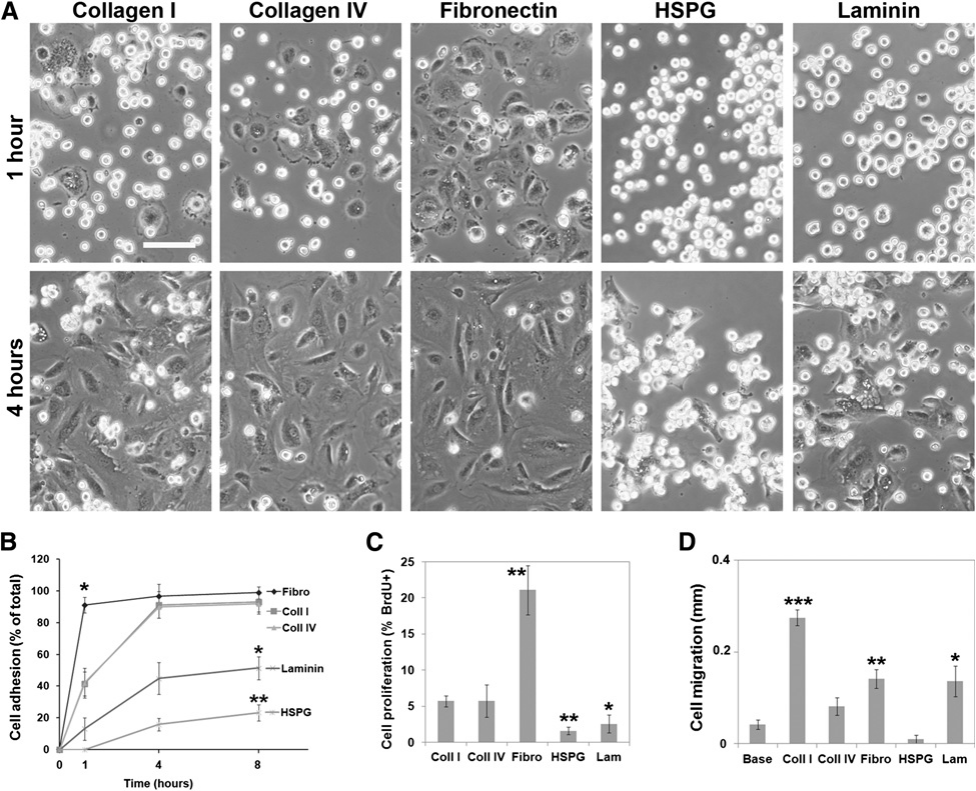
**Extracellular matrix (ECM) substrates differentially regulate cerebral pericyte behavior.** (**A**) Phase pictures showing pericyte adhesion and morphology on collagen I, collagen IV, fibronectin, HSPG, and laminin-1 after 1 and 4 hours. Scale bar = 100 μm. Note that pericytes attached well to fibronectin and the collagens but very poorly to HSPG. (**B**) Time course of pericyte adhesion to different ECM substrates over 8 hours. Adhesion assays were performed as described in the text, and all points represent the mean ± SEM of four experiments. Note that after 1 hour, the number of adherent pericytes was >90% on fibronectin, approximately 40% on collagens, approximately 10% on laminin-1, but less than 1% on HSPG. * *P*<0.01, ** *P*<0.001. (**C**) Influence of ECM substrates on pericyte proliferation. Proliferation assays were performed as described in the text, and all points represent the mean ± SEM of four experiments. Note that fibronectin strongly promoted (** *P*<0.01), whereas laminin-1 (* *P*<0.05) and HSPG (** *P*<0.01) inhibited pericyte proliferation. (**D**) Influence of ECM substrates on pericyte migration. Migration assays were performed as described in the text, and all points represent the mean ± SEM of four experiments. Note that pericyte migration was promoted most strongly by collagen I (*** *P*<0.001) and to a lesser degree by fibronectin (** *P*<0.01) and laminin-1 (* *P*<0.02).

First, the pericyte adhesion characteristics were investigated by performing adhesion assays at time-points of 1, 4, and 8 hours. A clear hierarchy among the ECM proteins quickly became evident (Figure 1A). After 1 hour, the pericytes were fully attached to fibronectin, and almost half the cells were also attached to collagen I and IV, but cells on HSPG and laminin-1 appeared rounded up and poorly attached. At the 4-hour time-point, the pericytes on fibronectin and the collagens were fully attached and spread, and the cells on laminin-1 had increased their attachment and had begun to spread whereas the cells on HSPG were still predominantly phase-bright unattached cells. The adhesion kinetic curves (Figure 1B) show that after 1 hour, fibronectin had promoted significantly more pericyte adhesion than any other substrate (91 ± 4.9% compared with 41.5 ± 7.6% on collagen I, *P*<0.01), with only 13 ± 7.2% cell attachment to laminin-1, and less than 1% of adhesion to HSPG. After 8 hours, fibronectin and collagen I and IV had all promoted greater than 90% adhesion of pericytes, whereas the pericyte adhesion to laminin and HSPG was significantly less (fibronectin 98.8 ± 3.8 versus laminin 51.3 ± 7.3 (*P*<0.01) and HSPG 29.2 ± 5.2 (*P*<0.001)). Thus, there was a clear hierarchy in the strength of adhesion of pericytes for different vascular ECM substrates in the order: fibronectin > collagens > laminin-1 > HSPG.

Next, the influence of different ECM components on pericyte proliferation and migration was investigated. Pericyte proliferation was examined by BrdU incorporation. Pericytes were cultured in serum-free conditions on the different substrates, then BrdU was added to cells for 3-hours, followed by BrdU immunofluorescence (IF) detection. Pericyte proliferation was promoted most strongly by fibronectin (Figure 1C), with a proliferation rate four-fold greater than any other substrate (21.1 ± 3.4% on fibronectin compared with 5.7 ± 0.8% on collagen I, *P*<0.01). Collagen IV also supported pericyte proliferation (5.7 ± 2.2%). However, compared with collagen I (5.7 ± 0.8%), both laminin-1 (2.6 ± 1.2%; *P*<0.05) and HSPG (1.6 ± 0.5%; *P*<0.01) significantly inhibited pericyte proliferation.

The influence of the ECM substrate on pericyte migration was investigated by using the scratch assay. Pericytes were first grown to confluence in serum-containing PCM and cultured on ECM-coated 24-well plates, then horizontal and vertical scratches were made to the monolayer to produce linear regions devoid of cells. The medium was then switched to serum-free N1 medium, and migration was measured over the next 16 hours. Pericyte migration was most effectively promoted by collagen I (0.28 ± 0.02 mm versus 0.04 ± 0.01 mm on uncoated plastic, *P*<0.001), followed by 50% lower levels on fibronectin (0.14 ± 0.02 mm, *P*<0.01) and laminin-1 (0.14 ± 0.03 mm, *P*<0.02) (Figure 1D and Additional file 1: Figure S1). Compared with uncoated plastic (baseline), cells on HSPG showed an anti-migratory trend, although this failed to reach statistical significance. Taken together, these results demonstrate that specific ECM substrates have markedly different effects on pericyte behavior. Consistent with its upregulation during cerebrovascular remodeling and its regenerative influence on other cell types [21,22,29], fibronectin supports pericyte remodeling by strongly promoting pericyte proliferation and migration. Both collagen I and IV also support pericyte proliferation, but the two collagens have differential effects on pericyte migration, with collagen I having a much stronger effect. Although laminin-1 is only a weak promoter of pericyte adhesion and proliferation, it does support migration. Most strikingly, HSPG appears to be non-permissive for all aspects of pericyte behavior, suggesting that, within intact blood vessels, HSPG restricts pericyte proliferation and migration, thus preventing excessive and unwanted vascular remodeling.

### Cerebral pericytes express a limited repertoire of integrins

**Figure 2:**
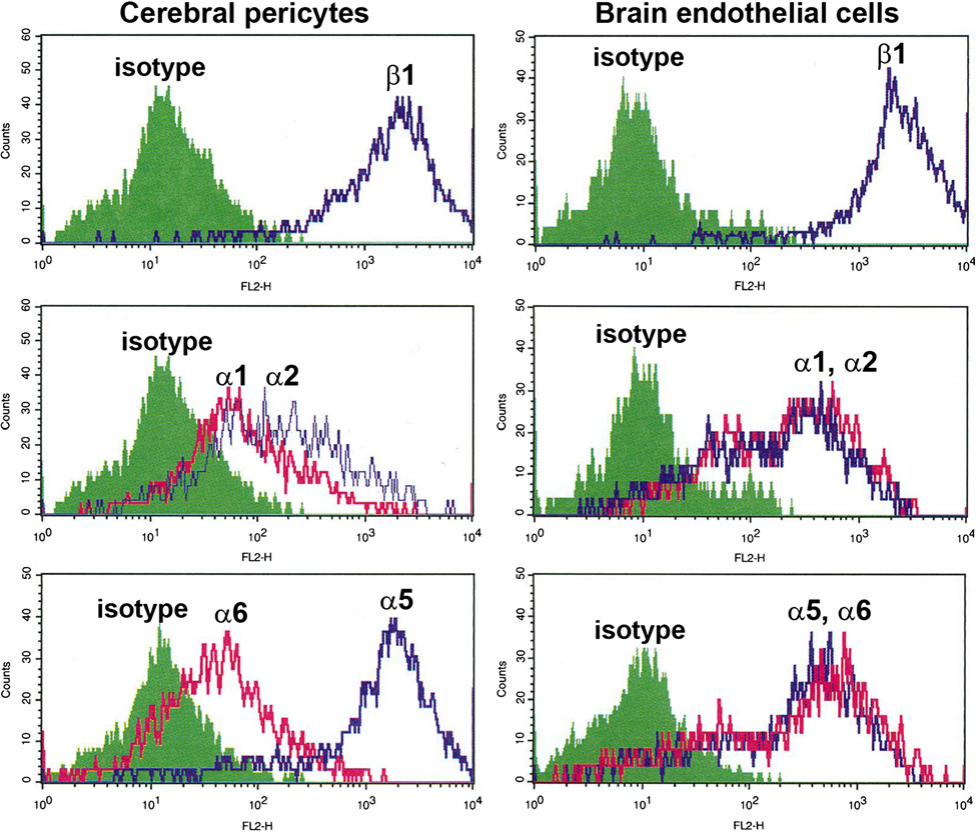
**Characterization of β1 integrin expression in brain pericytes and brain endothelial cells (BECs).** Cells were cultured on collagen I for 2 days, and then expression of integrin subunits was analyzed by flow cytometry. Note that in contrast to BECs, which expressed equivalent levels of the α5 and α6 subunits, and relatively high levels of α1 and α2 subunits, pericytes expressed high levels of α5, but much lower levels of the α1, α2, and α6 subunits.

Integrins are the major class of cell-surface receptors that mediate effects of the ECM [19,20]. To characterize the integrin-expression profile of cerebral pericytes, flow cyometry was performed (Figure 2). This showed that pericytes express several different β1 integrins, including α1 and α2 (collagen/laminin receptors), α5 (fibronectin), and α6 (laminin) integrins, but pericytes do not express the α4 integrin subunit (data not shown). Significantly, of all the β1 integrin subunit partners, α5 had by far the highest expression level, with much lower levels of the α1, α2, and α6 integrin subunits (α5:α6 ratio of 20:1). By comparison, BECs express equivalent levels of the α5 and α6 integrin subunits, and higher levels of the α1 and α2 subunits. This may explain why pericytes adhere strongly to fibronectin, but only weakly to laminin-1.

### Tumor necrosis factor-α promotes a pro-modeling pericyte phenotype

**Figure 3:**
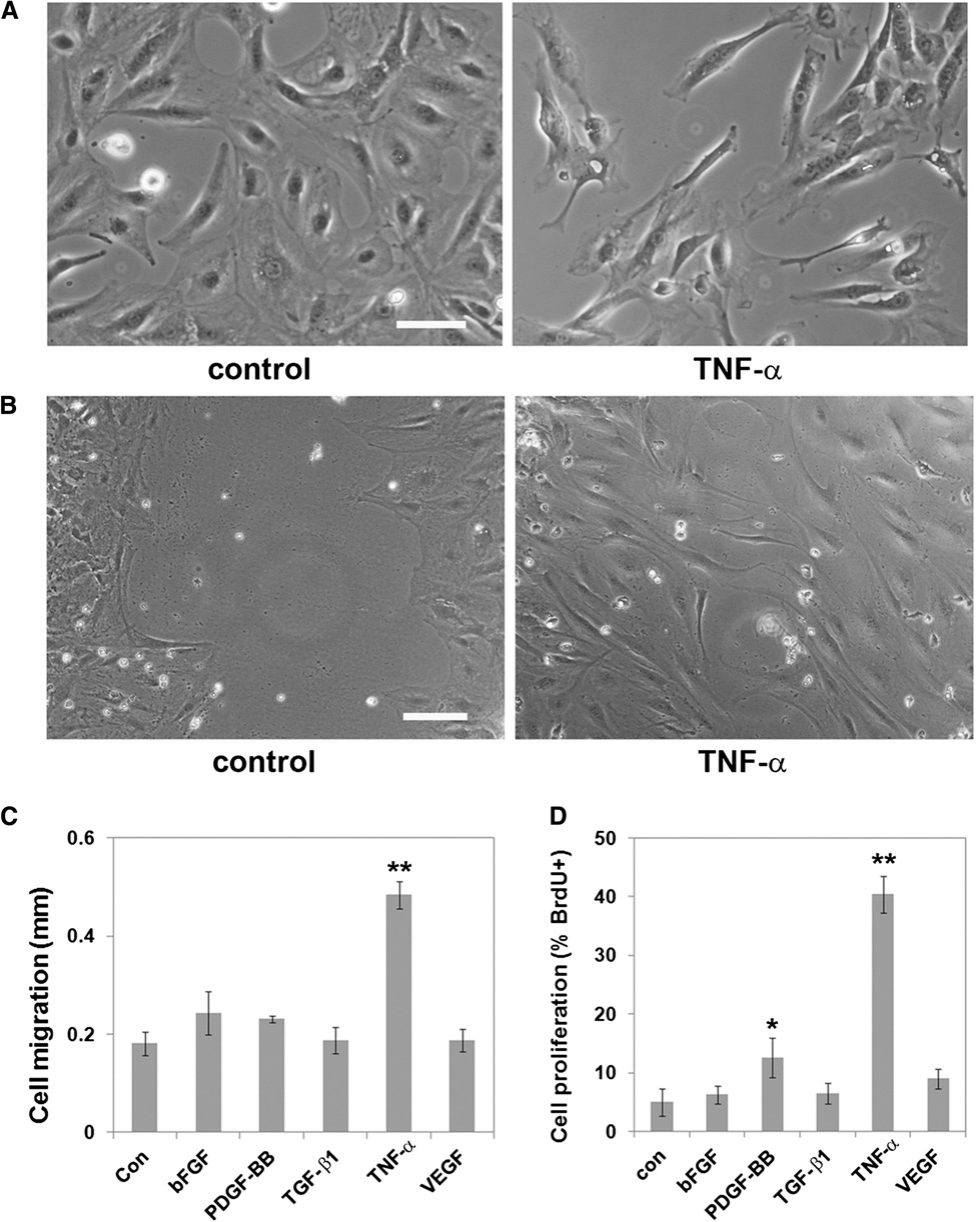
**Tumor necrosis factor (TNF)-α strongly promotes a pericyte remodeling phenotype.** (**A**) The influence of TNF-α on pericyte morphology. Scale bar = 50 μm. Note that TNF-α promoted a switch in pericyte morphology, from a well-spread, rhomboid phenotype into a predominantly bipolar, polarized morphology. (**B**) The influence of TNF-α on pericyte migratory capacity. Scale bar = 100 μm. Note that TNF-α enhanced pericyte migration, as illustrated by a rapid closing of the scratch defect. (**C**,**D**) Influence of cytokines on (**C**) pericyte migration and (**D**) proliferation. Assays were performed as described in the text, and all points represent the mean ± SEM of four experiments. Note that pericyte migration and proliferation were promoted most strongly by TNF-α * *P*<0.05, ** *P*<0.01).

Vascular remodeling is promoted by a number of different growth factors, including VEGF, bFGF, and PDGF-BB [10,30,31]. Cytokines including TGF-β and TNF-α also influence this process [32,33]. To investigate how these factors influence pericyte remodeling, we examined their effect on pericyte migration and proliferation. These studies showed that of all the factors tested, TNF-α had the most dramatic effect on pericyte behavior. TNF-α altered pericyte morphology from a well-spread, rhomboid phenotype of the control cells into a predominantly bipolar, polarized morphology (Figure 3A). Furthermore, this TNF-α-induced switch in morphology correlated with an increased migration rate. The untreated (control) pericytes migrated slowly as a wave of well-spread rhomboid-shaped cells, still displaying a significant gap between the opposing migrating borders, whereas TNF-α-treated cells migrated much faster as a wave of bipolar, elongated cells, which at the same time-point had almost closed the gap in the monolayer (Figure 3B). Quantification of this effect demonstrated a strong pro-migratory influence of TNF-α (0.48 ± 0.03 mm versus 0.18 ± 0.02 mm under control conditions, *P*<0.01) (Figure 3C). Furthermore, BrdU incorporation assays (Figure 3D) showed that TNF-α was also highly effective at stimulating pericyte proliferation (40.4 ± 3.1% for TNF-α versus 5.0 ± 2.4% for control cells, *P*<0.01). Thus, TNF-α strongly promotes cerebral pericyte remodeling behavior, stimulating both pericyte proliferation and migration.

### Cerebral pericyte integrin expression is regulated by cytokines

**Figure 4:**
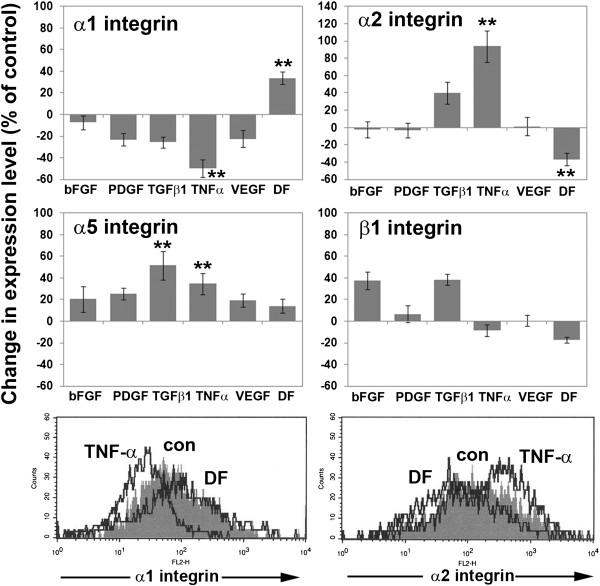
**Influence of cytokines on pericyte expression of β1 integrins.** (**A**) Pericytes were cultured on collagen I in the presence of different cytokines as described in Methods. After 2 days of culture, pericyte expression of the α1, α2, α5, α6, and β1 integrin subunits was analyzed by flow cytometry, and the expression level is shown as the percentage change relative to control conditions (no cytokine). All points represent the mean ± SEM of four experiments. Note that tumor necrosis factor (TNF)-α promoted a switch in integrins, downregulating α1 while strongly upregulating α2 integrin, and this effect was exactly the opposite to that seen when pericytes differentiated (DMEM + FBS; DF condition). In addition, several factors, including transforming growth factor (TGF)-β1 and TNF-α increased α5 integrin expression (** *P*<0.01). (**B**) Histogram flow-cytometry plots illustrating the contrasting influence of TNF-α and DF on pericyte expression levels of α1 and α2 integrin subunits. Note that TNF-α increased the α2:α1 ratio, whereas DF had the opposite effect.

It is now well established that growth factors and cytokines exert some of their effects on cell behavior by modulating ECM-integrin interactions [34,35]. To address whether this occurs in pericytes, we investigated whether growth factors and cytokines influence integrin expression by pericytes. Pericytes were cultured in PCM on collagen I-coated six-well plates in the presence of different factors for 2 days, before their integrin-expression levels were quantified by flow cytometry. Analysis of the ability of each factor to increase or decrease pericyte expression of given integrin subunits identified two obvious effects (Figure 4). First, TNF-α promoted a switch in collagen-binding integrins, concomitantly downregulating α1 (by 49.8 ± 8.3% of control, *P*<0.01), while strongly upregulating α2 integrin (193.9 ± 18.3% of control, *P*< 0.01). Interestingly, this TNF-α-induced switch from α1 to α2 integrin was exactly the opposite to that which occurred when pericytes were induced to differentiate (indicated by increased expression of α-smooth muscle actin) by culture in DMEM containing 10% FBS (DF). In this system, DF increased α1 expression (to 133.6 ± 5.8% of control, *P*<0.01), but decreased α2 integrin (by 36.7 ± 7.5% of control, *P*<0.01). Thus, TNF-α and DF exert antagonistic effects on pericyte remodeling status; TNF-α promotes pericyte transition into a remodeling phenotype, which correlates with a switch from α1 to α2 integrins, whereas DF has the opposite effect. Second, several factors increased pericyte expression of the pro-angiogenic α5 integrin subunit. TGF-β1 was the most effective at promoting α5 integrin expression (to 151.6 ± 13.4% of control, *P*<0.01), and TNF-α also promoted this effect (increasing α5 levels to 134.6 ± 9.6% of control, *P*<0.01). Pericyte α6 integrin-expression levels were not significantly altered by any of the factors tested.

### Cerebral pericytes use predominantly α2 integrin to attach to collagens

**Figure 5:**
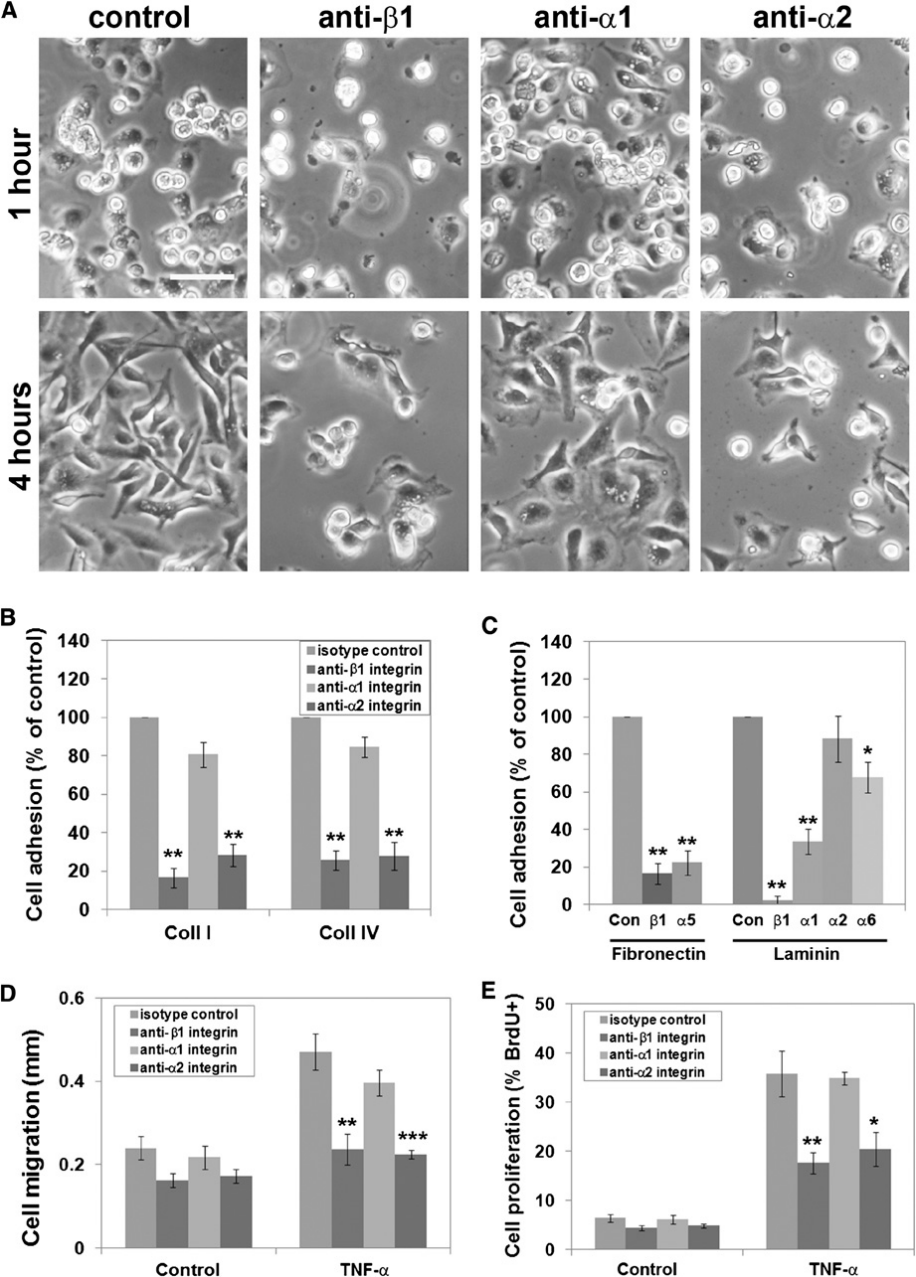
**Identifying roles for specific β1 integrins in regulating pericyte behavior.** (**A**) Pericyte adhesion to collagen I is mediated by the α2β1 integrin. Phase pictures showing that pericyte adhesion to collagen I was blocked by antibodies specific for β1 and α2 subunits but not by the anti-α1 integrin antibody. Scale bar = 50 μm. (**B**) Quantification of integrin blockade studies. Adhesion assays examining pericyte adhesion to collagen I and collagen IV were performed as described in the text, and all points represent the mean ± SEM of four experiments. Note that pericyte adhesion to collagen I and IV was blocked by antibodies specific for β1 and α2 subunits but not by the anti-α1 integrin antibody (** *P*<0.001). (**C**) Identifying pericyte integrins that mediate adhesion to fibronectin and laminin-1. Note that α5β1 integrin mediated pericyte adhesion to fibronectin, whereas adhesion to laminin-1 was mediated primarily by α1 and to a lesser degree by α6 integrin (* *P*<0.01, ** *P*<0.001). (**D,E**) Examining the role of α2 integrin in pericyte migration (**D**) and proliferation (**E**). Assays were performed as described in the text, and all points represent the mean ± SEM of four experiments. Note that pericyte migration and proliferation on collagen I was significantly blocked by antibodies against the β1 or α2 integrin subunits, but was not affected by the anti-α1 integrin antibody (* *P*<0.05, ** *P*<0.02, *** *P*<0.01).

Our studies reveal that TNF-α promotes transformation of pericytes into an active remodeling phenotype, and that this correlates with a switch in expression of collagen-binding integrins, from α1 to α2. So what is the functional significance of this switch in β1 integrins? One possibility is that upregulation of α2 integrin confers on pericytes additional adhesive or signaling properties, facilitating increased adhesion to basal lamina collagen and increasing migratory and mitotic capacity. In support of this hypothesis, previous studies have shown that α2 integrin has a much higher affinity than α1 integrin for the ECM substrate collagen I [36,37], raising the possibility that TNF-α-induced upregulation of α2 integrin might enhance pericyte adhesion to collagen I, and thus increase migration and proliferation. To test this idea, we first examined the relative contribution of α1 and α2 integrins in pericyte adhesion to collagen I and collagen IV. Adhesion was quantified in the presence of well-characterized function-blocking monoclonal antibodies directed against the β1 (Ha2/5), α1 (Ha31/8) or α2 (Ha1/29) integrin subunits. Pericyte adhesion to collagen I was significantly inhibited by monoclonal antibodies against the β1 and α2 integrin subunits, but anti-α1 antibodies had no significant effect (Figure 5). This is apparent in the phase-contrast pictures (Figure 5A) which show that antibodies against β1 and α2 integrins, but not α1 integrin, blocked pericyte adhesion at the 1-hour time-point, and also substantially inhibited pericyte cell spreading at the 4-hour time-point. Pericyte adhesion to collagen I in 1- hour adhesion assays was inhibited by anti-β1 (to 16.5 ± 5.0% of control, *P*<0.001) and anti-α2 (to 28.3 ± 5.8% of control, *P*<0.001) antibodies, and pericyte adhesion to collagen IV was also inhibited by anti-β1 (to 25.8 ± 5.1% of control, *P*<0.001) and anti-α2 (to 27.8 ± 7.1% of control, *P*<0.001) antibodies, whereas the anti-α1 antibodies had no obvious effect (Figure 5B). These results show that pericytes use the α2β1 integrin to attach to both types of collagen. In addition, we also investigated which integrins mediate pericyte adhesion to fibronectin and laminin-1 (Figure 5C). The 1-hour adhesion assays showed that pericyte adhesion to fibronectin was effectively blocked by antibodies against β1 (to 16.5 ± 5.6% of control, *P*<0.001) and α5 (to 22.3 ± 6.4% of control, *P*<0.001) integrins, indicating that α5β1 is the major pericyte receptor responsible for adhesion to fibronectin. Similar experiments showed that pericyte adhesion to laminin-1 was almost totally blocked by anti-β1 antibody (to 2.3 ± 2.2% of control, *P*<0.001) and partially blocked by anti-α1 (to 33.5 ± 6.7% of control, *P*<0.001) and anti-α6 (to 67.8 ± 8.1% of control, *P*<0.01) antibodies, but was not significantly affected by the anti-α2 antibody, indicating that pericyte adhesion to laminin-1 is mediated by a combination of α1β1 and α6β1 integrins.

### The pro-modeling influence of tumor necrosis factor-α is blocked by α2 integrin-blocking antibodies

Because TNF-α promotes parallel increases in pericyte remodeling status and α2 integrin expression, we next examined whether α2β1 integrin mediates some of the change in pericyte behavior, by measuring pericyte migration and proliferation on collagen I under the influence of TNF-α, in the presence of integrin-blocking antibodies. TNF-α significantly promoted pericyte migration and proliferation compared with controls, and the pro-modeling effects of TNF-α were significantly blocked by antibodies against the β1 (migration reduced from 0.47 ± 0.04 mm to 0.24 ± 0.04 mm, *P*<0.02; proliferation reduced from 35.8 ± 4.7% to 17.6 ± 2.2%, *P*<0.02) or α2 (migration reduced from 0.47 ± 0.04 mm to 0.23 ± 0.01 mm, *P*<0.01; proliferation reduced from 35.8 ± 4.7% to 20.4 ± 3.4%, *P*<0.05) integrin subunits, but were not significantly affected by the anti-α1 integrin antibody (Figure 5D,E). This demonstrates that TNF-α stimulates pericyte migration and proliferation through a α2 integrin-dependent mechanism.

### Pericyte α2 integrin is induced during cerebrovascular remodeling *in vivo*

**Figure 6:**
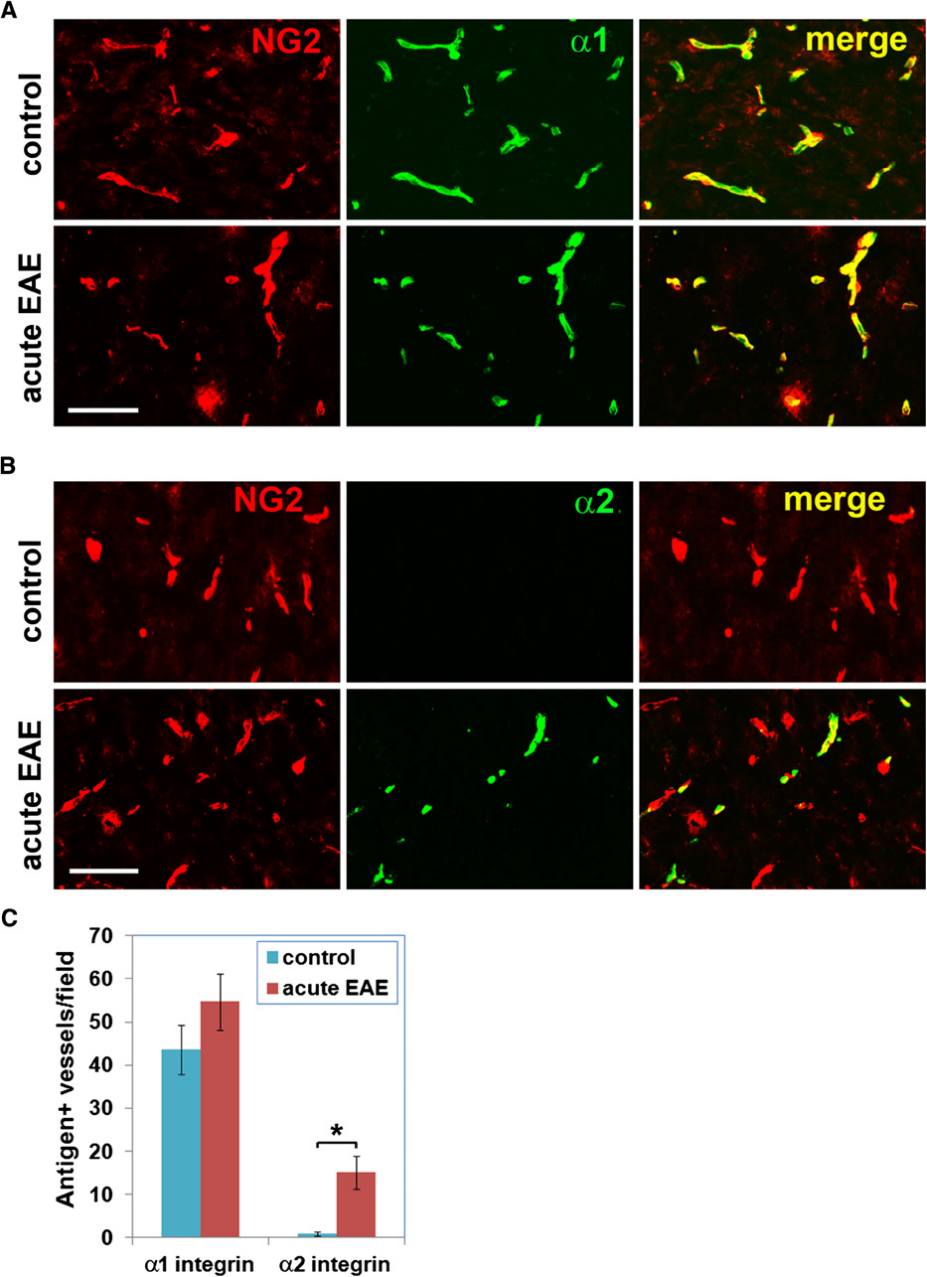
**Pericyte α2 integrin is induced during cerebrovascular remodeling *in vivo*.** Pericyte expression of (**A**) α1 and (**B**) α2 integrins (both Alexa Fluor-488, green) was analyzed by dual immunofluorescence (IF) with the pericyte marker NG2 (Cy3, red) in frozen brain sections taken from mice with acute experimental autoimmune encephalomyelitis (EAE) or control mice. Scale bar = 50 μm. (**C**) Quantification of this data. Each point represents the mean ± SEM of three different experiments. Note that α1 integrin strongly co-localized to NG2-positive pericytes, both in control and acute EAE tissue, whereas α2 integrin expression was undetectable on cerebral vessels in control tissue, but showed strong induction on pericytes in the acute EAE brain. *P*<0.001.

To determine whether pericyte expression of α1 or α2 integrins is altered during cerebrovascular remodeling *in vivo*, we examined these events in the brains of mice with EAE, an animal model of multiple sclerosis, in which marked vascular remodeling occurs [38,39]. Using NG2 to identify pericytes [40-42], dual IF showed that α1 integrin had a vascular pattern that strongly co-localized to NG2-positive pericytes, with no appreciable difference in the vascular intensity level of α1 integrin between control and acute EAE tissue (Figure 6A). Interestingly, α2 integrin expression was undetectable on cerebral vessels in control tissue, whereas some vessels in acute EAE brain tissue showed marked induction of α2 integrin expression (15 ± 3.8 vessels/field compared with 0.9 ± 0.5 vessels/field in control tissue, *P*<0.001), and this strongly co-localized to NG2-positive pericytes (Figure 6B,C). This demonstrates that pericytes in remodeling vessels in the brain of EAE mice show strong induction of α2 integrin.

## Discussion

Pericytes have an extremely close relationship with the ECM components of the basal lamina of blood vessels [3,7]. In this study, we took an *in vitro* approach to determine how different vascular ECM substrates influence pericyte adhesion, migration, and proliferation, and to define the integrin receptors that mediate these effects. We then examined the interplay between cytokines and ECM–integrin interactions in regulating pericyte behavior. Our studies showed that fibronectin and collagen I promote pericyte proliferation and migration, whereas the proteoglycan HSPG had an overall inhibitory influence on pericytes. Of the cytokines tested, TNF-α had the strongest pro-modeling influence, stimulating pericyte proliferation and migration, concomitantly triggering a marked switch in pericyte integrins, from α1 to α2 integrin, the exact opposite to that seen in differentiated pericytes. Inhibition studies showed that α2 integrin mediates pericyte adhesion to collagen I and IV, and function blockade of α2 integrin prevented the pro-modeling influence of TNF-α. To our knowledge, these are the first studies to demonstrate that ECM constituents are a major influence on pericyte remodeling. Specifically, they suggest a model in which HSPG restricts pericyte remodeling in stable vessels, but during inflammation, TNF-α triggers a switch in pericyte integrins, from α1 to α2, thereby promoting pericyte proliferation and migration on collagen. These studies thus identify a fundamental molecular mechanism that mediates pericyte transformation into an active remodeling phenotype.

### The extracellular matrix regulates pericyte functions

Several factors that regulate pericyte behavior including PDGF-BB and VEGF, are known [10,43], although surprisingly, the influence of ECM components has not been directly addressed. In this study, we found that fibronectin and collagen I drive cerebral pericytes towards a pro-modeling phenotype, which is in keeping with the influence of these ECM proteins on other cell types. Fibronectin is a strong promoter of endothelial cell proliferation and migration [29,44], and is a strong promoter of vascular remodeling under different conditions including development, tumor-associated neovascularization, and hypoxia-induced cerebrovascular remodeling [21,23,45,46]. Likewise, collagen I promotes angiogenic endothelial remodeling both *in vitro* and *in vivo*[47,48]. Our finding that fibronectin and collagen I also stimulate pericyte remodeling suggests that endothelial cells and pericytes use common mechanisms to switch from a quiescent stable phenotype into an active remodeling one. In stark contrast, we found that the proteoglycan HSPG was non-permissive for all aspects of pericyte behavior, consistent with the finding that HSPG inhibits mesangial adhesion to fibronectin [49]. Our data suggest that within stable cerebral blood vessels, HSPG might restrict pericyte proliferation and migration, thus preventing unwanted vascular remodeling. These results are consistent with the idea that the positive/negative balance of ECM cues may play an important role in determining vascular remodeling status.

### Tumor necrosis factor-α strongly promotes a pericyte remodeling phenotype

Evidence suggests that TNF-α promotes vascular remodeling *in vivo*. Exogenous TNF-α was shown to promote angiogenic sprouting in the rat cornea and the chick chorioallantoic membrane [50]. In a mouse model of airway inflammation, TNF-α and endothelial expression of TNF receptor 1 (TNF-R1) were increased, and inhibition of this pathway blocked remodeling [32]. At the cellular level, TNF-α promotes endothelial cell proliferation [27], migration, and tube formation [50]. In the current study, we found that TNF-α also promoted pericyte proliferation and migration, consistent with recent data that TNF-α stimulates cerebral pericyte migration and matrix metalloproteinase-9 production [51]. Together, these observations support a fundamental role for TNF-α in mediating vascular remodeling.

### Switching of β1 integrins by remodeling pericytes

A major finding to emerge from this study is that the pro-modeling influence of TNF-α correlated with a switch in pericyte expression of β1 integrins, from α1 to α2, whereas differentiating pericytes showed the opposite switch. A similar switch has been described on chondrocytes [52]. So what might be the functional significance of this switch? Although α1 and α2 integrins show great similarity in their sequence homology [19], some clear functional differences between these two integrins have been reported. First, the ligand specificity of α1 and α2 integrins seems to be cell-type-specific. Glomerular epithelial cells (GECs) use α2β1 to attach to collagen, and use both α1β1 and α2β1 to attach to laminin, whereas renal mesangial cells use both α1β1 and α2β1 to attach to collagen, but use only α1β1 to adhere to laminin [53,54]. Second, compared with α1β1, α2β1 integrin has much higher affinity for collagen I [36,37], implying that α2 integrin expression confers on cells an increased adhesion and signaling capability on this substrate.

Several studies have highlighted an important role for α2β1 integrin in promoting cell proliferation and migration in other systems. PDGF-B-induced proliferation and migration of vascular smooth muscle cells is blocked by function-blocking anti-α2 antibodies or enhanced by the α2β1 integrin agonist aggretin [55,56]. Furthermore, many studies have described an important role for α2β1 integrin in promoting the migration and/or metastatic spread of tumor cells, including melanoma [57], and carcinoma cells of the colon, prostate, liver, and mammary gland [58-61]. In another study, TNF-α conferred an invasive transformed phenotype on mammary epithelial cells that was accompanied by increased α2 integrin expression, and specific blockade of α2 integrins inhibited this transformation [62]. This TNF-α-induced transformation bears a remarkable similarity to our own findings with pericytes, suggesting the presence of a common fundamental mechanism by which TNF-α stimulates cell migration through a α2 integrin-dependent mechanism.

An angiogenic role for α2β1 integrin in endothelial cells has been well described [63,64]. A recent study by Stratman *et al*. defined an important role for pericytes in stimulating endothelial basement membrane formation and vessel maturation, but also demonstrated a requirement for α2 integrin in the early stages of tube formation, [65]. Taken with our own findings, this is consistent with the notion that α2β1 integrin provides pro-angiogenic signals, both in endothelial cells and pericytes during the early stages of vessel remodeling. So how essential is α2 integrin for these events? Interestingly, although α2 integrin knockout (KO) mice are viable and fertile, they exhibit defective branching morphogenesis in mammary epithelial ducts [66]. In future experiments, we plan to test whether α2 integrin plays a similar role in vessel sprouting by examining cerebrovascular remodeling in α2 integrin KO mice, both during EAE and in a mouse model of chronic mild hypoxia.

## Conclusions

The aim of this study was to determine how ECM components present in the vascular basal lamina influence pericyte remodeling behavior, and how cytokines regulate these events. Fibronectin and collagen I promoted pericyte proliferation and migration, but the proteoglycan HSPG had an inhibitory influence on pericyte behavior. The pro-inflammatory cytokine TNF-α strongly promoted pericyte proliferation and migration, and concomitantly induced a switch in pericyte integrins, from α1 to α2 integrin, the opposite to that seen when pericytes differentiate. Inhibition studies showed that α2 integrin mediates pericyte adhesion to collagens, and that function blockade of α2 integrin inhibited the pro-modeling influence of TNF-α. Together, these results suggest a model in which ECM constituents influence pericyte remodeling status. In this model, HSPG restricts pericyte remodeling in stable vessels, but during inflammation, TNF-α triggers a switch in pericyte integrins, from α1 to α2, which stimulates pericyte proliferation and migration on collagen. These results thus define a fundamental molecular mechanism by which TNF-α stimulates pericyte remodeling in an α2 integrin-dependent manner.

## Competing interests

The authors declare that they have no competing interests.

## Authors’ contributions

UT prepared the pericyte cultures, performed the functional assays and flow cytometry, and contributed to drafting the manuscript. AB prepared the cell cultures, performed the IF analysis of EAE tissue, and contributed to drafting the manuscript. JVW-A prepared the endothelial cell cultures, performed some IF analysis of EAE tissue, and contributed to drafting the manuscript. RM conceived of the study, performed flow cytometry, and drafted the manuscript. All authors read and approved the final manuscript.

## Supplementary Material

Additional file 1: Figure S1. TNF-α promotes cerebral pericyte remodeling *in vitro*, via a switch from α1 to α2 integrins. (PDF 414 kb) Click here for file
