# Supplementary material for: TNF-α promotes cerebral pericyte remodeling in vitro, via a switch from α1 to α2 integrins
Source: J Neuroinflammation. 2013 Mar 1;10:33. doi: 10.1186/1742-2094-10-33 (PMC3616978; doi:10.1186/1742-2094-10-33)

Supplemental data section for Tigges et al

TNF-α promotes cerebral pericyte remodeling in vitro, via a switch from α1 to α2 integrins

Supplemental Figure 1.

Extracellular matrix substrates differentially regulate pericyte migration. Migration assays were performed as described in Materials and Methods. Scale bar = 100µm. Note that pericyte migration was most strongly promoted by collagen I, as illustrated by a rapid closing of the scratch defect.

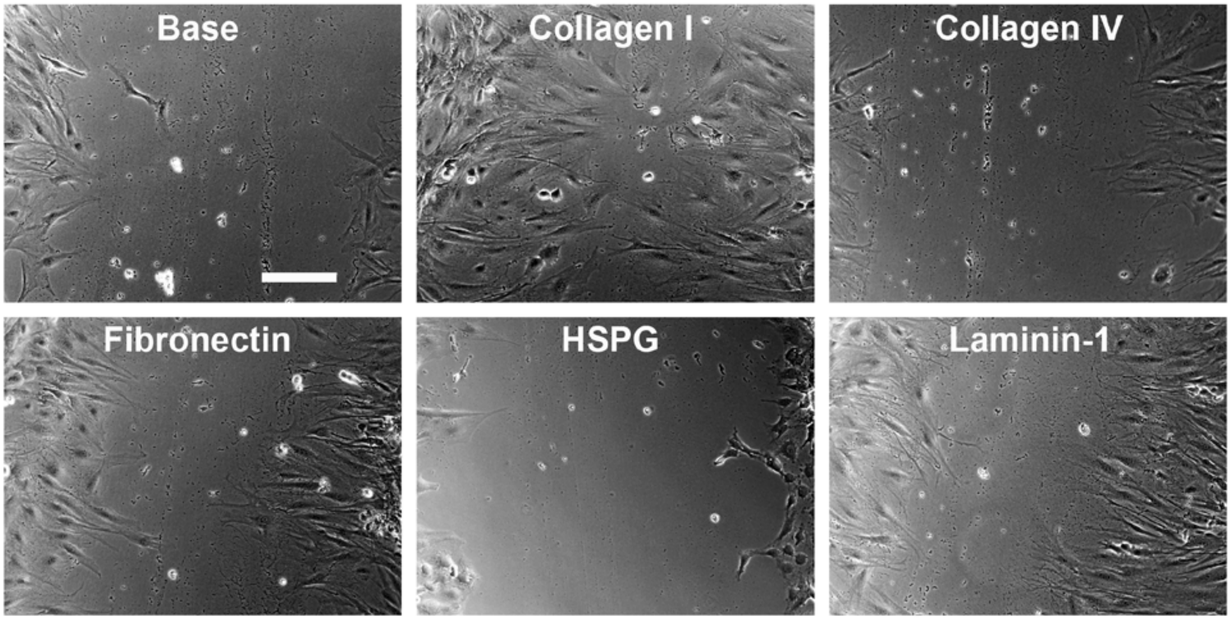

Supplement: Additional file 1: Figure S1. — TNF-α promotes cerebral pericyte remodeling in vitro, via a switch from α1 to α2 integrins. (PDF 414 kb) [file 1742-2094-10-33-S1.pdf]
